# European training requirements in Neonatology 2021—towards a unified training standard for Neonatologists

**DOI:** 10.1038/s41390-025-03840-5

**Published:** 2025-01-31

**Authors:** Charles C. Roehr, Sven Wellmann, Tomasz Szczapa, Pascal Fentsch, Petra Hüppi, Olivier Baud, Ana Alarcon, Willem P. de Boode, Michael Hall, Olivier Danhaive, Maximo Vento

**Affiliations:** 1 European Society for Paediatric Research, Satigny, Switzerland; 2 European Board of Neonatal & Child Health Research, Satigny, Switzerland; 3 National Perinatal Epidemiology Unit, Clinical Trials Unit, Oxford Population Health, University of Oxford, Oxford, UK; 4 Southmead Hospital, Women’s and Children’s, Newborn Care, North Bristol NHS Trust, Southmead, Bristol, UK; 5 Faculty of Health and Life Sciences, University of Bristol, Bristol, UK; 6 Department of Neonatology, University Children’s Hospital Regensburg (KUNO), Hospital St. Hedwig of the Order of St. John, University of Regensburg, Regensburg, Germany; 7 Department of Neonatology, Neonatal Biophysical Monitoring and Cardiopulmonary Therapies Research Unit, Poznan University of Medical Sciences, Poznan, Poland; 8 Division of Development and Growth, Department of Paediatrics, Gynaecology and Obstetrics, Geneva University Hospitals, Geneva, Switzerland; 9 Obstetrical Perinatal and Pediatric Epidemiology Research Team, EPOPE, French Institute for Medical Research and Health, Université Paris Cite, CRESS, INSERM, INRAE, Paris, France; 10 Department of Neonatal Medicine, Cochin Port-Royal Hospital, FHU PREMA, AP-HP Centre, Paris, France; 11 University of Geneva, Geneva, Switzerland; 12 Department of Neonatology, Hospital Sant Joan de Déu and Hospital Clínic; BCNatal (Barcelona Centre for Maternal, Foetal and Neonatal Medicine); Institut de Recerca Sant Joan de Déu, Barcelona, Spain; 13 Department of Surgery and Medical-Surgical Specialties, Faculty of Medicine and Health Sciences, Universitat de Barcelona, Barcelona, Spain; 14 Neonatology, Radboudumc Amalia Children’s Hospital, Radboud University Medical Center, Nijmegen, The Netherlands; 15 University of Southampton, School of Health Sciences, Southampton, UK; 16 Department of Paediatrics, St-Luc University Hospital, Catholic University of Louvain, Brussels, Belgium; 17 Department of Pediatrics, Benioff Children’s Hospital, University of California San Francisco, San Francisco, CA USA; 18 Division of Neonatology, University and Polytechnic Hospital, La Fe (HULAFE), Valencia, Spain

## Abstract

**Abstract:**

The European Society for Paediatric Research (ESPR) first developed recommendations for a Neonatology specific European training curriculum in 1998, with updates in 2007 and 2021. The aim of these recommendations was to define a common, European standard of training for national educational programmes for Neonatologists. Following the Union of European Medical Specialists’ (UEMS) framework of European Training Requirements (ETR), and similar to the American Board of Pediatrics (ABP) recommendations, graduates of training programmes conforming to the ETR will be eligible throughout Europe for recognition of equality of training, and with that should be enabled to freedom-of-movement. This concept also accounts for neonatal specialists. We therefore present the pan-European work on the ETR Neonatology in its third iteration (ETR III), summarising the basic requirements for contemporary training programmes, trainers, and training centres in neonatology. We highlight the European School of Neonatology (ESN) as a comprehensive online educational platform which provides the theoretical and practical background to satisfy the ETR-III. Lastly, we introduce the European Board of Neonatal & Child Health Research (EBNCHR) as a committee dedicated to gaining acceptance for the concept of harmonising education and training in Neonatology and recognising Neonatology as a Paediatric subspecialty in every European Union member state.

**Impact:**

- Neonatology currently is not uniformly recognised as a Paediatric subspecialty throughout the 27 European countries. Hence, training in Neonatology formerly followed no commonly agreed standard throughout the European Union (EU).
- To ensure a minimum standard of care, an agreed minimum standard of training is required. The European Society for Paediatric Research (ESPR) has led on generating an EU-accredited, pan-European Syllabus for Neonatal training in Europe, the European Training Requirements (ETR) in Neonatology (2021).
- This article presents the ETR Neonatology from commissioning to accreditation and discusses means of how high-grade post-graduate education, aligned with the ETR can be achieved by practitioners.

## Background

Neonatology, as the youngest sub-specialty in Paediatrics, was first mentioned by Shafer in 1963.^1^ In just 60 years, practitioners of neonatal medicine have made enormous advances leading to dramatic improvements in the care of one of the most fragile patient groups, babies, mothers, and their families.^2^ As a profession, Neonatology has achieved remarkable improvements in reducing morbidity and mortality by embracing a culture of openness, inclusivity and a multidisciplinary team approach with continuous knowledge exchange which places the patient and their families at the heart of its practice.^3^ On the shop-floor, meticulous clinical acumen, an in-depth understanding of fundamental physiologic principles, detailed patient observation and an early adoption of rigorous scientific study, including embracing the principles of evidence-based medicine and quality improvement, were the backbone of ongoing improvements in care.^4–8^ Thus, contemporary neonatal medicine encompasses the care for healthy and compromised newborn babies, including perinatal consultation, newborn stabilisation, care of complex genetic, metabolic, and endocrine conditions, pre- and postoperative care for infants with surgical conditions of all organs as well as acutely ill term to extremely preterm infants, - often cared for in highly technologized environments, and, not infrequently, requiring compassionate palliative care.^9–13^ Consequently, practitioners of neonatal medicine need to be attuned to recognising the complex needs of the parents and families of newborn infants as a whole, and care for these emphatically in often culturally complex settings.^14^

Drastic improvements in the care for the newborn and their families over the short course of 6 decades stand in strong contrast to regional differences in availability and quality of neonatal care^15,16^; and striking differences in accessibility to health care, disparities in resource allocation, education and, last not least, physicians’ access to specific knowledge resources and postgraduate training still account for significant disparities in patient outcomes.^17–19^ The European Unions’ (EU) “Free movement of persons” directive was designed to encourage EU citizens to exercise their right to move and reside freely within the Member States and encourages them to mutually recognise professional diplomas, certificates, and other evidence of formal qualifications, also for medical professionals.^20^ Consequently, the EU commissioned a taskforce, the Union of European Medical Specialists (UEMS), to harmonise training of the EU’s paediatric workforce.^21^ The UEMS recognises that high-quality postgraduate training programmes are indispensable for achieving high standards of patient care and, by forming the European Board of Paediatrics (EBP), the Paediatric Section of UEMS, endeavoured to optimise training programmes in Paediatrics and recognised subspecialties throughout the European Union (EU).^22^ Thus, since 1993, completion of UEMS recognised training allows doctors to practise medicine throughout the EU. In the spirit of fairness and equitable resource allocation and utilisation, it is therefore fundamental for specialist doctors to receive equivalent, high-quality training in their home country, in each of the EU member states, to prevent migration for educational purposes.^23^

## Generating The European Syllabus For Training In Neonatology

For Neonatology, the disparity in availability, duration, and accreditation of European Neonatology specific training programmes has been documented by Dr Obladen, formerly president of the European Society for Paediatric Research (ESPR).^24^ Acknowledging previous attempts by the EBP to standardise training requirements within Europe, and in line with Obladen, Drs Stiris, Carnielli, Greisen and Marlow, postulated on behalf of the ESPR, that conforming training and accreditation throughout Europe would be an enormous asset to improve the quality of neonatal care provided throughout the whole European region, decrease infant mortality, increase scientific productivity, and help facilitate subspecialist mobility.^25^ They devised the first two editions of the European Training Requirements (ETR) in Neonatology, which were issued in 1998 and 2007, respectively.^25^ These earlier ETR editions were already aligned with recommendations from several European National Training Authorities (NTAs) and the recommendations of the European Common Trunk Syllabus in Paediatrics, the CESP/UEMS.^22,23^

In the following, we present the journey of the updated, 3rd edition of the ETR (ETR III), defining the current common standard for core Neonatal Training in Europe.^26^ The 2021 ETR III in Neonatology (henceforth referred to as ETR Neonatology 3.0) comprises a curriculum and assessment framework which may be utilised to:

- Harmonise training in Neonatology between different European countries;
- Establish clearly defined standards of knowledge and skills required to practise Neonatology at a tertiary care level (see Appendix 1);
- Foster the development of a European network of proficient tertiary care centres for Neonatology (see Appendix 2) and enable freedom-of-movement for neonatal specialists; and
- Enhance European contributions to international scientific progress in the field of Neonatology.

Consequently, the ETR-Neonatology 3.0 defines the contemporary aims of training, the content and the duration of the training programme, the basic requirements for entering such a programme and a spectrum of required qualifications for training centres and tutors for practising Neonatology at specialist level within the EU.

In 2018, the UEMS tasked the ESPR with updating the ETR Neonatology to account for the many medical and technological advances in Neonatology, as well as growing consumer involvement in care decisions and expectations on common EU care standards. Based on the ETR-II,^25^ other national European syllabi, and the European Standards of Care for Newborn Health,^27,28^ ETR Neonatology 3.0 was drafted by Drs Roehr, Szczapa and Vento as officers of the European Society for Paediatric Research (ESPR) and the European Board of Neonatal & Child Health Research (EBNCHR). Following this, representatives from all 27 European country’s national neonatal societies/NTAs, and members of international parent organisations were offered to review and comment on the ETR Neonatology 3.0. An online, two rounded modified Delphi process was used, in which we aimed for at least 75% group agreement, after written feedback from participants. Any remaining inconsistencies were discussed and full consented in an in-person meeting (Maastricht, September 2019), to ensure alignment with regional and national requirements.

## Syllabus subcommittees, consultation, and accreditation

The ETR Neonatology 3.0 was produced between 2018 and 21. A first draft was presented to representatives of the European Academy of Paediatrics (EAP) and the EBP. Following their review and after incorporating feedback from different stakeholders (as outlined above), including the European Foundation for the Care of the Newborn Infant (EFCNI), the ETR Neonatology 3.0 was consented by the representatives of all 27 European national neonatal societies/NTAs. Following this consensus, the ETR Neonatology 3.0 was approved by the EAP Secondary-Tertiary Care Council, and, following their recommendation, was formally ratified by the EAP Secondary-Tertiary Care Council and the EAP General Assembly in January 2021. As the final confirmative step, full UEMS approval was granted in April 2021 and the ETR Neonatology 3.0 was published online by UEMS^29^ (see also Appendix 1). Further, to illustrate the progress made since publication of the ETR II, Table 1 shows the unique features of the ETR Neonatology 3.0 (Table 1). The complete process from commissioning to accreditation of the ETR Neonatology 3.0 was published elsewhere.^30^ For transparency and in accordance to the Journal Pediatric Research’s data availability policy, as outlined on their website. Materials described in this manuscript, including all relevant data, will be made freely available to any researcher wishing to use them for non-commercial purposes, upon reasonable request to the corresponding author, without breaching participant confidentiality.

**Table 1:** Unique features of the ETR Neonatology 3.0

Unique features of the ETR Neonatology 3.0	
Pan-European Standardisation	ETR 3.0 aims to establish a uniform standard for neonatal training across the EU. It addresses the lack of consistent quality care and seeks to ensure that all neonatal professionals meet a high-level standard of competence.
Facilitated Mobility Across Borders	ETR 3.0 helps facilitate that neonatal specialists can have their qualifications recognised throughout the EU, simplifying cross-border career mobility.
Inclusive Development	ETR 3.0 has been developed through a collaborative process by high-level experts in neonatal medicine and research, incorporating feedback from EU member states’ national neonatal societies as well as international parent organisations. In this way the curriculum acknowledges diverse regional needs and specificities.
Competency-Based Education	ETR 3.0 incorporates Competency-Based Medical Education (CBME) and Entrustable Professional Activities (EPAs), emphasising the need for the practical application of skills in real-life medical environments.
Commitment to High Standards	ETR 3.0 is regularly updated to reflect the latest advancements in neonatal medicine, maintaining its position at the forefront of neonatal training and ensuring that practitioners are equipped with current best practices.
Comprehensive Training Framework	ETR 3.0 offers a robust and integrated training framework that combines theoretical and practical education through the European School of Neonatology (ESN). This includes educational initiatives such as:
	The clinically-integrated Master of Advanced Studies (MAS) in Neonatology.
	An academically-focused Master of Science (MSc) in Neonatology (NOTE programme) offered in collaboration with the University of Southampton.
	Special Courses and Training covering a wide range of theory, skills, and competencies.
	The Neonatologist Performed Echocardiography (NPE) training programme and governance structure.
	The free 16-part lecture series ‘Perspectives on Effective Neonatology’.
Global Relevance:	ETR 3.0 serves as a model for neonatal education worldwide, with potential applications in other regions.

## Discussion and future directions

The ETR Neonatology 3.0 was commissioned in recognition of the need for multi-national training recommendations for practitioners in neonatal medicine in a fast-changing environment of rapid medical and technological advances, as well as growing consumer involvement in care decisions and expectations on common EU care standards. We believe that the multifaceted and inclusive approach in generating the ETR Neonatology 3.0 ensures that knowledge of every aspect of modern neonatal care required to deliver high-quality, current day neonatal medicine, has been met. The ETR Neonatology Version 3.0 very much focuses on practical knowledge transfer into practice of competence profiles (knowledge, skills and attitudes), where the majority of teaching, learning and assessment takes place in a real-life, medical environment. By integrating the principles of Competency-Based Medical Education (CBME) and Entrustable Professional Activities (EAPs), placing professionalism, competence, and trust at the heart of The Syllabus, with outcome-based approaches to the development, realisation and review of educational initiatives, the highest standards for contemporaneous, effective professional education have been met.^31–34^

Appreciatively, with over 30 years since the first ETR Neonatology was devised to help standardise neonatal training and accreditation in Europe it may be bemoaned that there still is neither a EU-wide accepted national training programme for neonatology, nor have all EU-member states agreed on recognising neonatology as a Paediatric subspecialty. However, as Breindahl M et al. have shown, the first two versions of the ETR have helped with standardisation and harmonisation of subspecialist neonatal training in Europe.^35^ Nonetheless, there were still significant barriers to overcome, including ongoing intra-European inconsistencies regarding the organisation and accreditation of specialty and subspecialty training at regional and national level. As an example, in Germany medical (sub-)specialists’ accreditation is managed by the medical chambers of the 16 federal states. In their recently consented and now enacted, new educational regulation (2023) they address core cognitive and methodological competencies as well as action competencies and manual skills in line with the ETR Neonatology as the minimum standard for neonatal sub-specialist training. The minimum training time for sub-specialist’ training in Germany is currently 24 months following completed specialty training in paediatrics, which lasts a minimum of five years. Availability of sufficient training opportunities in high-volume tertiary neonatal centres are quoted as reasons for the deviation of the ETR recommended training times. Consequently, German neonatal training units are required by the medical chambers to provide evidence of caring for a critical mass of neonatal cases, and a sufficient case mix, before being recognised as sub-specialty training centres. In other European countries, for example in Poland, neonatology is recognised as a paediatric subspecialty, but the Polish national specialty programme is currently not aligned with the ETR, despite clear recommendations from the Polish Neonatal Society. Economic pressures on the healthcare system and diminishing resources for teaching, training and accreditation are quoted as reasons for shorter training times. This to illustrate how, despite the endorsement and good will by the regional national societies to implement the ETR Neonatology 3.0, more is needed to achieve its implementation. Challenges include the need for closer collaboration of NTAs, policy makers and governments, on regional, national and international level. Nonetheless, we believe the EU-wide uptake of the ETR Neonatology 3.0 is well achievable. The EBNCHR already holds detailed discussions with the representatives of the 27 EU NTAs at national and federal levels, alongside discussions with the relevant chambers of EU member states. We are confident that with time, the ETR Neonatology 3.0 (or one of its further iterations) will be widely endorsed as the EU standard of neonatal education and training. Meanwhile, the EBNCHR, the ESN and the ESPR, together with the NTAs are working out the complex governance system. For this, a unified system for documenting neonatal training and accreditation of high-quality training centres and trainers is ultimately required. With its multiple online training opportunities, the ESPR provides ample resources with contemporary high-quality content (Table 2).

**Table 2:** Important links to resources around the ETR Neonatology 3.0

Important links	
ETR-3	https://www.espr.eu/activities-projects/etr_neonatology.php
ESN website	https://esn-education.org/
ESN on the ESPR website	https://www.espr.eu/activities-projects/esn.php
ESN course page	https://esntutorial.espr.eu/eduMarket
ESN Campus platform	https://esncampus.isyflow.ch
ESN Tutorial platform	https://esntutorial.espr.eu
NOTE website	https://moodle.neonataltraining.eu/
NOTE on the ESPR website	https://www.espr.eu/activities-projects/note.php
EBNCHR page	https://www.espr.eu/activities-projects/ebnchr.php
ESN LinkedIn page	https://www.linkedin.com/company/european-school-of-neonatology
ESPR-ESN Bluesky	https://bsky.app/profile/espr-esn.bsky.social
ESPR-ESN X page	https://x.com/ESPR_ESN

Outside of Europe, other national or federal health care systems tackle similar challenges in standardising postgraduate education, healthcare provision and accreditation. In the USA, for instance, national training programmes have been devised by the NTAs with a focus on regional recognition of equivalence of training, allowing freedom of movement between regions.^36–38^ For Europe, the ESPR will continue to work together with NTAs from the EU to improve the ETR Neonatology to maintain it at the cutting edge of neonatal practice and education. In the coming years the EPA framework will be refined together with the national societies/ NTAs and by gathering data through ESPRs educational initiatives. This dataset will then provide the foundation for the next revision of the ETR Neonatology, so that it can be updated and restructured according to EPA principles.

## Professional education & training aligned with the Etr Neonatology 3.0

Since the 1960’s, the ESPR has been at the forefront of efficiently delivering neonatology specific educational content in Europe. For over a decade, the online Neonatal Online Training and Education (NOTE) programme, delivered in collaboration between the ESPR and the University of Southampton, UK, provides one of the highest quality postgraduate neonatal education programmes within the global Neonatal Virtual Learning Environment.^39^ Successful NOTE participants have the option of receiving academic credit at Master level (see below list of links to resources). To further meet the continuously growing demand for speciality specific training, without necessarily resulting in a higher-education diploma, the ESPR has recently formed the European School of Neonatology (ESN).^40^ The ESN provides a pan-European, comprehensive and interprofessional training framework for neonatal doctors, and in the future also advanced nurse practitioners, involved in the care for newborn babies. The modular ESN curriculum in Neonatology provides a structured and standardised approach at the height of current day standards in neonatal training and postgraduate education.^41^ Based on the training requirements defined in The Syllabus, comprehensive and evidence-based training programmes, incorporating the diverse theoretical and practical aspects of neonatal medicine ensures that graduates of the ESN training modules are well-equipped for delivering a common level of safe and effective care. Completion of the full ESN curriculum allows for the acquisition of a Master of Science (MAS). Links to NOTE, the ESN MAS, and several other ESPR educational opportunities are listed in Table 2.

## Supplementary information

Supplemental information

Supplemental information

## Data Availability

No data was handled. Interested readers are welcome to request further information on the process of generating the ETR III, as well background information on the European legislation for specialist training from the corresponding author.
