# Supplementary material for: European training requirements in Neonatology 2021—towards a unified training standard for Neonatologists
Source: Pediatr Res. 2025 Jan 31;98(3):848–52. doi: 10.1038/s41390-025-03840-5 (PMC12507670; doi:10.1038/s41390-025-03840-5)
Supplement: Supplementary file 2 — Supplemental information [file 41390_2025_3840_MOESM2_ESM.pdf]

Appendix 2 - Requirements for training institutions

**Perinatal Unit Size**	Neonatology training centres should have sufficient patient numbers, ideally handling a wide range of neonatal diseases. Recommended is a minimum of 40 very low birth weight infants (<1500g) admitted per year, with trainees expected to manage resuscitation and primary care for at least 25 of these, including 10 extremely low birth weight infants (<1000g).
**Obstetrics Integration**	Training hospitals should be part of a perinatal centre, equipped for prenatal diagnosis, high-risk pregnancy management, and neonatal resuscitation during deliveries.
**Specialty Access**	Training units should have access to various paediatric specialties, including surgery, cardiology, respiratory medicine, radiology, and more. Access to perinatal/paediatric pathology for necropsy is also required.
**Supporting Staff**	Neonatal units must have trained supporting staff (e.g. radiographers, pharmacists, and social workers) to assist in newborn care. Administrative support should be available to reduce the workload on medical staff.
**Neonatal Nursing Staff**	Senior nurses with neonatal experience and those responsible for in-service training are essential. Nurse-to-patient ratios should be manageable, with one-to-one nursing required during critical situations.
**Medical Staff Requirements**	Hospitals should have at least three accredited neonatologists, with 24-hour coverage by trained neonatal medical staff. There should be two tiers of resident staff providing continuous bedside supervision.
**Parental Involvement**	Parents should be encouraged to participate in care and decision-making. Facilities should be available for breastfeeding, including electric breast pumps and milk banks, along with support services like social workers, bereavement counsellors, and psychological advisors.
**Transport Services**	Hospitals should facilitate prenatal maternal transfers to avoid postnatal transfers of sick infants. Neonatal transport services must be adequately staffed and equipped.
**Equipment Standards**	Training units must have a budget and policy for equipment maintenance and upgrades, including incubators, ventilators, monitors, and other essential neonatal care tools. Access to 24-hour laboratory services is crucial.
**Quality Assurance**	Written protocols for medical and nursing staff should be in place, regularly reviewed, and include procedures for resuscitation and extremely preterm infant management. Monitoring systems for morbidity and mortality, with annual data reports, are necessary.
**Assessment of Training Centres**	National regulation governs the assessment of training centres and trainers, aligning with European standards and the UEMS-accredited syllabus.
